# The Proceedings of the International Hahnemannian Association

**Published:** 1893-03

**Authors:** 


### HOMŒOPATHIC PHYSICIAN,

A MONTHLY JOURNAL OF

HOMŒOPATHIC MATERIA MEDICA AND CLINICAL MEDICINE.

"If our school ever gives up the strict inductive method of Hahnemann, we are lost, and deserve only to be mentioned as a caricature in the history of medicine."—CONSTANTINE HERING.

Vol. XIII. MARCH, 1893. No. 3.

### EDITORIAL.

THE PROCEEDINGS OF THE INTERNATIONAL HAHNEMANNIAN ASSOCIATION have now been presented to our readers in such detail that only a small part remains unpublished. A fair idea is therefore obtainable by those who diligently peruse these pages of the exceedingly interesting and valuable character of these meetings. Perhaps one of the most profitable of the discussions was that relating to the treatment of animals which appeared in the February number.

These creatures not gifted with imagination cannot be accused of being influenced toward recovery by any subtle mental impressions. They therefore conspicuously illustrate the effect of administering the simillimum and realizing a true cure.

This point so constantly urged, alike by editors and contributors, in the pages of this journal, is as constantly forgotten or ignored by the majority of the practitioners claiming to belong to our school. Yet it is the one thing that will make the new school a success, and give its votaries that power and influence which they so much covet, and which most of them are so far from attaining.

By their fruits shall they be known. The ability to cure means power, and that attribute is respected by all mankind.

Our materia medica, though defective, yet contains a golden wealth of "indications" for the happy control of the most deplorable "sick conditions." To avail ourselves of this wealth we must lay aside all preconceived notions, all prejudices and partialities, and diligently study the pathogenetic records, and seek to find in them a close similar to the case of sickness which we strive to cure.

But this advice is not followed by very many of our faith. Prominent men there are among us who reject our materia medica, and in their zeal to correct its defects seek to tear it up by the roots. They fill the journals with denunciations of its imperfections; they propose that the gigantic task of constructing a reliable materia medica shall be begun all over again, and even offer a scheme of revised materia medica which so confuses the student that it is not wonderful so many of our professed adherents abandon it altogether and practice a system of "adjuvants" which does not distinguish them from the average physician of the old school.

To all these wanderers the proceedings of the Internationals are most wholesome reading, and must tend to recall them to the straight path of pure Homœopathy. We are not sorry, therefore, we have occupied so much of our valuable space with these papers.

